# Association between the red blood cell distribution width-to-albumin ratio (RAR) and cardiovascular disease: A cross-sectional analysis of NHANES 2003 to 2016

**DOI:** 10.1097/MD.0000000000046351

**Published:** 2026-05-12

**Authors:** Siqi Ma, Qu Jin, Zhuo Yan, Jingyi Yang, Liping Chang

**Affiliations:** a Changchun University of Traditional Chinese Medicine, Changchun City, Jilin Province, China; b Changchun University of Traditional Chinese Medicine Affiliated Hospital, Changchun City, Jilin Province, China.

**Keywords:** cardiovascular disease, cardiovascular disease risk, cross-sectional study, National Health and Nutrition Examination Survey, red blood cell distribution width and albumin ratio

## Abstract

The purpose of this study was to investigate the association between red blood cell distribution width and albumin ratio (RAR) and cardiovascular disease (CVD) and to determine the relevant thresholds. Researchers acquired data from the NHANES database between 2003 and 2016. Restricted cubic spline curve (RCS) analysis and multivariate logistic regression were conducted. The objective is to better understand how RAR influences CVD risk and potential nonlinear interactions. This study’s sensitivity analysis included subgroup and interaction testing. The present study is predicated on the analysis of 10,909 individuals. Logit analysis has identified RAR as a separate CVD risk factor. When RAR was analyzed using quartiles, after accounting for potential confounders, RAR2, RAR3, and RAR4 were significantly linked to a higher risk of CVD, *P* < .001 for all. The RCS analysis revealed a nonlinear association between RAR and CVD. Subsequent to the execution of subgroup and interaction analyses, a consistency was demonstrated by all subpopulations in this study in relation to the overall population. To summarize, there is a nonlinear association between RAR and CVD among US adults. Adjusted modeling revealed that a RAR value below 0.416 was a critical threshold at which the odds ratio was very high at 9771.155 (95% confidence interval: 1491.836–63,998.653, *P* < .001), indicating a strong association with CVD. The tool under discussion has been demonstrated to facilitate clinicians in the assessment of the likelihood of cardiovascular events being predicted. RAR has been demonstrated to be a simple, effective and inexpensive method of predicting CVD.

## 1. Introduction

Cardiovascular disease (CVD) is a leading cause of mortality and morbidity worldwide and has been associated with a significant increase in both disease incidence and mortality rates in recent years. This trend has emerged as a critical public health concern that poses a substantial threat to human health. Epidemiological data indicate that CVD accounts for over 17 million deaths annually, highlighting its profound impact on global health outcomes.^[1]^ Despite the substantial advancements achieved in the field of cardiovascular medicine, it is imperative to recognize the persistent challenges that remain unresolved. The primary aim of this study is to provide healthcare professionals with novel diagnostic and therapeutic strategies for CVDs. This research underscores the significance of exploring emerging biomarkers and risk factors in order to enhance the early detection and management of these conditions.^[2]^ Accurate prediction and identification of CVD risk factors are crucial for facilitating early disease prevention and improving patient outcomes.

Red cell distribution width (RDW) serves as a biomarker reflective of erythrocyte heterogeneity and is intricately associated with systemic inflammatory processes.^[3]^ Recent findings have indicated a correlation between this and the prevalence and mortality of several inflammatory diseases, In addition to the foregoing diabetes,^[4]^ this text is about chronic obstructive pulmonary disease (COPD),^[5,6]^ stroke,^[7]^ and cancer.^[8]^ Albumin, on the other hand, has anti-inflammatory, antioxidant and antithrombotic properties,^[9]^ both of which are useful for CVD risk prediction, so the combined ratio of the 2 may provide a more comprehensive prognostic indicator.^[10]^ Existing studies have shown that red blood cell distribution width and albumin ratio (RAR) is associated with mortality in patients with CVD,^[11]^ heart failure,^[12]^ diabetes mellitus and related complications,^[13,14]^ stroke,^[15]^ and rheumatic diseases.^[16]^

The existing body of research predominantly focuses on the relationship between RAR and CVD mortality, with limited exploration of the association between RAR and CVD incidence independently. Furthermore, the underlying mechanisms linking RAR to CVD risk remain incompletely elucidated, thereby complicating the interpretation of current findings. This study endeavors to address these gaps by conducting a comprehensive analysis of the relationship between RAR and CVD risk. Through the utilization of large-scale datasets and rigorous statistical methodologies, we aim to provide a more definitive understanding of the prognostic significance of RAR in the context of CVD.

## 2. Materials and methods

### 2.1. Study population

Data for this cross-sectional study were derived from the National Health and Nutrition Examination Survey (NHANES) conducted between 2003 and 2016. The Centers for Disease Control and Prevention recorded, compiled, and uploaded the data to the National Center for Health Statistics. Detailed information regarding the NHANES survey methods and analytic guidelines can be accessed online at [NHANES Survey Methods and Analytic Guidelines] (https://wwwn.cdc.gov/nchs/nhanes/continuousnhanes/default.aspx) (accessed on March 1, 2025). The NHANES database is reviewed and managed by the National Center for Health Statistics Committee. Secondary analyses utilizing this dataset do not require additional ethical approval, as all participants provided voluntary informed consent. The primary NHANES website is available at [Centers for Disease Control and Prevention NHANES] (http://www.cdc.gov/nchs/nhanes.htm) (accessed on April 7, 2025).

**Figure 1.**
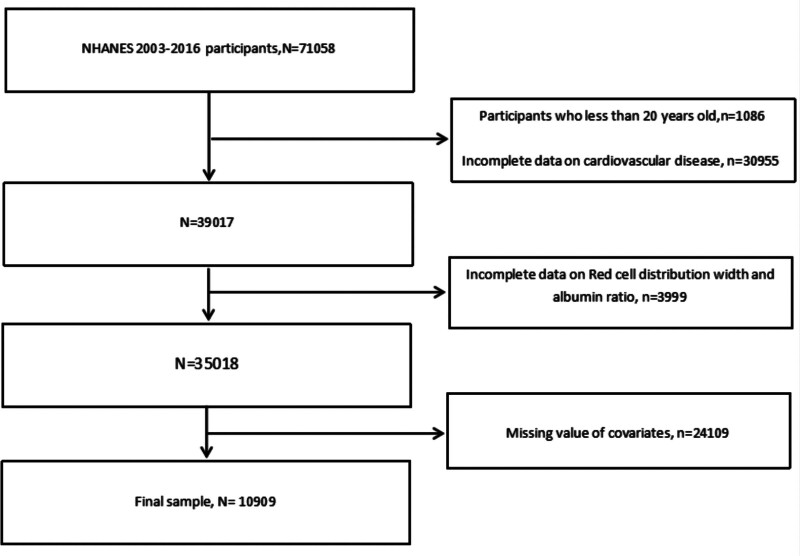
Flow chart of study participants. N = the number of patients being included, n = the number of patients being excluded.

Participants under 20 years of age and those with incomplete data for CVD, RAR, or relevant covariates were excluded from the analysis. A total of 71,058 participants completed interviews across the 14 survey cycles. Of these, 1086 participants under 20 years of age, 30,955 with missing CVD data, 3999 with missing RAR data, and 24,109 with incomplete covariate data were excluded. The final analytic sample comprised 10,909 participants. A detailed flowchart of the participant selection process is presented in Figure 1.

### 2.2. Variables

#### 2.2.1. CVD

In this study, the diagnosis of CVD was ascertained through self-reported physician diagnoses obtained during individual interviews using a standardized medical condition questionnaire. Participants were asked the following question: “Has a physician or other healthcare professional ever informed you that you have congestive heart failure, coronary heart disease, angina, myocardial infarction, or stroke?” A positive response to any of these conditions was considered indicative of CVD. The definitions of congestive heart failure, myocardial infarction, angina pectoris, and coronary artery disease were consistent with the corresponding disease descriptions provided in established medical literature.

#### 2.2.2. RAR

Using the formula: RDW (%)/albumin (g/L) = RAR. RAR levels: RAR1 (≤0.2923), RAR2 (0.2924–0.3146), RAR3 (0.3147–0.3447) and RAR4 (≥0.3448).

#### 2.2.3. Covariables

To account for potential confounders associated with CVD, a comprehensive set of covariates was extracted from the NHANES database. These covariates encompassed demographic characteristics (age, sex, ethnicity, educational attainment, marital status, and family poverty-to-income ratio (PIR)), preexisting medical conditions (COPD, diabetes mellitus, and hypertension), and laboratory parameters (urinary creatinine [mg/dL], globulin [g/dL], total cholesterol, alanine aminotransferase (ALT) [U/L], platelet count [1000 cells/μL], and mean platelet volume [fL]).

Participants were categorized by ethnicity into Mexican American, non-Hispanic White, non-Hispanic Black, and other races. Marital status was classified into 3 categories: single, married, and cohabitating. Educational attainment was divided into 3 levels: <9 years, 9 to 12 years, and more than twelve years. Household income was stratified into 3 groups based on the PIR: low (PIR ≤ 1.3), medium (PIR > 1.3 to 3.5), and high (PIR > 3.5). preexisting medical conditions, including COPD, diabetes mellitus, and hypertension, were identified through a questionnaire that asked participants whether a physician had previously diagnosed these conditions.

This study utilizes a publicly available dataset, with additional verification conducted to ensure data integrity. Categorical variables are presented as proportions, while continuous variables are reported as mean (standard deviation) or median (interquartile range), with ranges specified where relevant. For comparisons between groups, categorical variables were analyzed using 3 models. Model 1 included adjustments for age, sex, ethnicity, marital status, educational attainment, and household PIR. Model 2 further adjusted for COPD, diabetes mellitus, hypertension, body mass index (kg/m^2^), and waist circumference (cm). Model 3 incorporated the same variables as Model 2, with additional adjustments for laboratory parameters, including urinary creatinine (mg/dL), globulin (g/dL), total cholesterol, aspartate aminotransferase (AST) (U/L), ALT (U/L), mean platelet volume (fL), and platelet count (1000 cells/μL).

#### 2.2.4. A statistical investigation was conducted in order to obtain meaningful data

To identify discrepancies between baseline data, we employed the *t*-test for continuous variables and the χ^2^ test for categorical variables. Subsequently, restricted cubic spline curve (RCS) were utilized for linear evaluation. Inflection points were determined using logistic regression analysis, and the threshold effect was assessed through segmented logistic analysis. The identification of inflection points was achieved by employing the likelihood ratio test and the bootstrap resampling method. Multivariate logistic regression analyses were conducted to evaluate subgroup heterogeneity. The relationships between RAR and subgroups were rigorously examined using likelihood ratio tests. Every analysis was carried out. R 3.3.2, a statistical software program, was used (http://www.example.com, R Foundation, Shanghai, China). www.R-project.org and the free statistical software version 1.5.^[17]^

## 3. Results

### 3.1. The study population is composed of the following subjects

The total number of participants who completed the interview was 71,058. After exclusion, this cross-departmental study ultimately included 10,909 participants from NHANES between 2003 and 2016. The detailed inclusion and exclusion process is shown in Figure 1.

### 3.2. Baseline characteristics

Table 1 lists the baseline characteristics of the 10,920 patients stratified by RAR quartiles. Participants were categorized according to their RAR levels into RAR1, RAR2, RAR3, and RAR4. There were 2714, 2717, 2741, and 2748 individuals in each group, respectively. The mean age of all groups was 52.0 ± 16.5 and 4454 (40.8%) were female. All variables showed significant differences except for AST and interquartile range. Those with higher RAR were typically older, female, The subject is a married individual or cohabitee, Caucasian, with moderate educational attainment, with moderate household income, and had lower incidence of COPD, hypertension, diabetes mellitus, and stroke. Body mass index and waist circumference increased with increasing RAR category. Mean globulin levels were significantly higher in the RAR4 group (3.0 ± 0.5 g/dL) compared to the other groups. Total cholesterol levels were highest in the RAR1 group (198.4 ± 41.5 mg/dL). The ALT levels varied significantly among the RAR groups (*P* < .001). Platelet count and mean platelet volume also differed significantly among RAR groups (*P* < .001). There were significant differences between groups in ALT, platelet count, mean platelet volume, and urinary creatinine RAR. These data highlight the heterogeneity of patient characteristics, which may influence the distribution of risk and prognosis in patients with CVD.

**Table 1:** Baseline characteristics.

Variables	RAR					
	Total	RAR1 (≤0.2923)	RAR2 (0.2924–0.3146)	RAR3 (0.3147–0.3447)	RAR4 (≥0.3448)	*P*
Participants	10,920	2714	2717	2741	2748	
Age, Mean ± SD	52.0 ± 16.5	45.8 ± 16.0	51.7 ± 15.7	54.7 ± 16.3	55.9 ± 16.3	<.001
Gender, n (%)						
Male	6455 (59.2)	1925 (71)	1757 (64.8)	1524 (55.6)	1249 (45.5)	<.001
Female	4454 (40.8)	786 (29)	955 (35.2)	1217 (44.4)	1496 (54.5)	
Education level (yr), n (%)						
<9	1104 (10.1)	229 (8.4)	279 (10.3)	302 (11)	294 (10.7)	<.001
9–12	4774 (43.8)	1116 (41.2)	1132 (41.7)	1221 (44.6)	1305 (47.6)	
>12	5027 (46.1)	1366 (50.4)	1301 (48)	1216 (44.4)	1144 (41.7)	
Race/ethnicity, n (%)						
Non-Hispanic white	5794 (53.1)	1662 (61.3)	1470 (54.2)	1454 (53)	1208 (44)	<.001
Non-Hispanic black	2043 (18.7)	231 (8.5)	378 (13.9)	544 (19.8)	890 (32.4)	
Mexican American	1429 (13.1)	397 (14.6)	406 (15)	340 (12.4)	286 (10.4)	
Others	1643 (15.1)	421 (15.5)	458 (16.9)	403 (14.7)	361 (13.2)	
Marital status, n (%)						
Married or living with a partner	6731 (61.8)	1830 (67.6)	1773 (65.5)	1635 (59.8)	1493 (54.5)	<.001
Living alone	4161 (38.2)	879 (32.4)	933 (34.5)	1101 (40.2)	1248 (45.5)	
Family income, n (%)						
Low	2530 (23.2)	553 (20.4)	561 (20.7)	624 (22.8)	792 (28.9)	<.001
Medium	4748 (43.5)	1119 (41.3)	1144 (42.2)	1243 (45.3)	1242 (45.2)	
High	3631 (33.3)	1039 (38.3)	1007 (37.1)	874 (31.9)	711 (25.9)	
chronic obstructive pulmonary disease, n (%)						
Yes	432 (4.0)	51 (1.9)	84 (3.1)	127 (4.6)	170 (6.2)	<.001
No	10,458 (95.9)	2657 (98)	2626 (96.8)	2603 (95)	2572 (93.7)	
Diabetes, n (%)						
Yes	1401 (12.9)	193 (7.1)	271 (10)	364 (13.3)	573 (20.9)	<.001
No	9501 (87.1)	2517 (92.9)	2438 (90)	2376 (86.7)	2170 (79.1)	
Hypertension, n (%)						
Yes	1406 (12.9)	242 (8.9)	325 (12)	366 (13.4)	473 (17.2)	<.001
No	9503 (87.1)	2469 (91.1)	2387 (88)	2375 (86.6)	2272 (82.8)	
Body mass index (kg/m^2^), Mean ± SD	28.8 ± 6.5	26.6 ± 4.8	28.1 ± 5.5	29.4 ± 6.2	31.2 ± 7.9	<.001
Waist circumference (cm), Mean ± SD	100.3 ± 16.0	94.5 ± 13.5	98.7 ± 14.6	102.0 ± 15.6	105.7 ± 17.7	<.001
globulin (g/dL), Mean ± SD	2.9 ± 0.5	2.7 ± 0.4	2.8 ± 0.4	2.9 ± 0.4	3.0 ± 0.5	<.001
cholesterol (mg/dL), Mean ± SD	195.0 ± 43.2	198.4 ± 41.5	199.0 ± 43.3	194.4 ± 42.7	188.5 ± 44.4	<.001
Aspartate aminotransferase AST (U/L), Mean ± SD	26.6 ± 18.9	27.0 ± 14.7	26.6 ± 14.0	26.2 ± 18.6	26.7 ± 25.8	.393
Alanine aminotransferase alt (U/L), Mean ± SD	26.3 ± 24.7	28.3 ± 22.3	27.2 ± 18.6	25.9 ± 31.9	23.9 ± 24.0	<.001
Platelet count SI (1000 cells/µL), Mean ± SD	246.6 ± 67.7	244.5 ± 57.9	243.7 ± 60.8	244.4 ± 65.0	253.8 ± 83.3	<.001
Mean platelet volume (fL), Mean ± SD	8.1 ± 0.9	8.1 ± 0.9	8.1 ± 0.9	8.2 ± 0.9	8.2 ± 1.0	<.001
Creatinine, urine (mg/dL), Median (IQR)	112.0 (65.0, 168.0)	109.0 (62.0, 169.0)	113.0 (64.0, 166.0)	112.0 (66.0, 165.0)	114.0 (69.0, 171.0)	.03

RAR = red blood cell distribution width and albumin ratio.

### 3.3. Single-factor and multi-factor analysis

Univariate analysis demonstrated that, with the exception of globulin (g/dL), marital status, AST (U/L), ALT (U/L), and mean platelet volume (fL), all other covariates exhibited an association with CVD (Table 2). After controlling for relevant variables, RAR was examined using quartiles, the present study has demonstrated that RAR2, RAR3 and RAR4 have a significant association with an increased risk of CVD, with odds ratios (OR) of 1.54 (95% confidence interval (CI): 1.26–1.89), 2.64 (95% CI: 2.19–3.18), and 4.13 (95% CI: 3.45–4.94), all *P* < .001. Table 3 shows that, relative to the lowest quartile (RAR1), the fully adjusted odds of prevalent CVD rose in a dose-responsive manner (Model 3: RAR4 OR = 2.16, 95% CI: 1.76–2.66; *P*-trend < .001). Estimates were only modestly attenuated after sequential adjustment for sociodemographic, metabolic, and inflammatory covariates, indicating that elevated red-cell distribution width-to-albumin ratio is independently associated with increased cardiovascular risk.

**Table 2:** Correlation of covariates with cardiovascular disease risk.

Variables	OR (95% CI)	*P*_value	Variables	OR (95% CI)	*P*
Age (yr)	1.06 (1.05–1.06)	<.001	Diabetes, n (%)		
Gender, n (%)			Yes	1 (reference)	
Male	1 (reference)	<.001	No	0.26 (0.23–0.3)	<.001
Female	0.71 (0.63–0.8)		Hypertension, n (%)		
Education level (yr), n (%)			Yes	1 (reference)	
<9	1 (reference)		No	0.44 (0.38–0.5)	<.001
9–12	0.62 (0.53–0.74)	<.001	Body mass index (kg/m^2^), Mean ± SD	1.03 (1.02–1.04)	<.001
>12	0.48 (0.4–0.56)	<.001	Waist circumference (cm), Mean ± SD	1.03 (1.02–1.03)	<.001
Race/ethnicity, n (%)			globulin (g/dL), Mean ± SD	1.21 (1.07–1.36)	.002
Non-Hispanic white	1 (reference)		cholesterol (mg/dL), Mean ± SD	0.99 (0.99–0.99)	<.001
Non-Hispanic black	1 (0.87–1.15)	.989	Aspartate aminotransferase AST (U/L), Mean ± SD	1 (1–1)	.077
Mexican American	0.6 (0.49–0.73)	<.001	Alanine aminotransferase alt (U/L), Mean ± SD	1 (1–1)	.28
Others	0.65 (0.55–0.78)	<.001	Platelet count SI (1000 cells/µL), Mean ± SD	1 (1–1)	<.001
Marital status, n (%)			Mean platelet volume (fL), Mean ± SD	1.01 (0.95–1.08)	.666
Married or living with a partner	1 (reference)		Chronic obstructive pulmonary disease, n (%)		
Living alone	1.12 (1–1.25)	.053	Yes	1 (reference)	
Family income, n (%)			No	0.21 (0.17–0.25)	<.001
Low	1 (reference)		Mean platelet volume (fL), Mean ± SD	0.28 (0.08–0.98)	.046
Medium	1.03 (0.9–1.19)	.622	Creatinine, urine (mg/dL), Median (IQR)	1 (1–1)	<.001
High	0.63 (0.54–0.74)	<.001			

CI = confidence interval, OR = odds ratio.

**Table 3:** Relationship between erythrocyte distribution width and albumin ratio and cardiovascular disease.

Quartiles	OR (95% CI)								
	No.	Crude	*P*	Model 1	*P*	Model 2	*P*	Model 3	*P*
Red cell distribution width and albumin ratio (RAR)	2711								
RAR1 (≤0.2923)	2712	1 (Ref)		1 (Ref)		1 (Ref)		1 (Ref)	
RAR2 (0.2924–0.3146)	2741	1.54 (1.26–1.89)	<.001	1.54 (1.26–1.89)	<.001	1.54 (1.26–1.89)	<.001	1.54 (1.26–1.89)	<.001
RAR3 (0.3147–0.3447)	2745	2.64 (2.19–3.18)	<.001	2.64 (2.19–3.18)	<.001	2.64 (2.19–3.18)	<.001	2.64 (2.19–3.18)	<.001
RAR4 (≥0.3448)	10,909	4.13 (3.45–4.94)	<.001	4.13 (3.45–4.94)	<.001	4.13 (3.45–4.94)	<.001	4.13 (3.45–4.94)	<.001
Trend.test	2711		<.001		<.001		<.001		<.001

### 3.4. Nonlinear relationships

**Figure 2.**
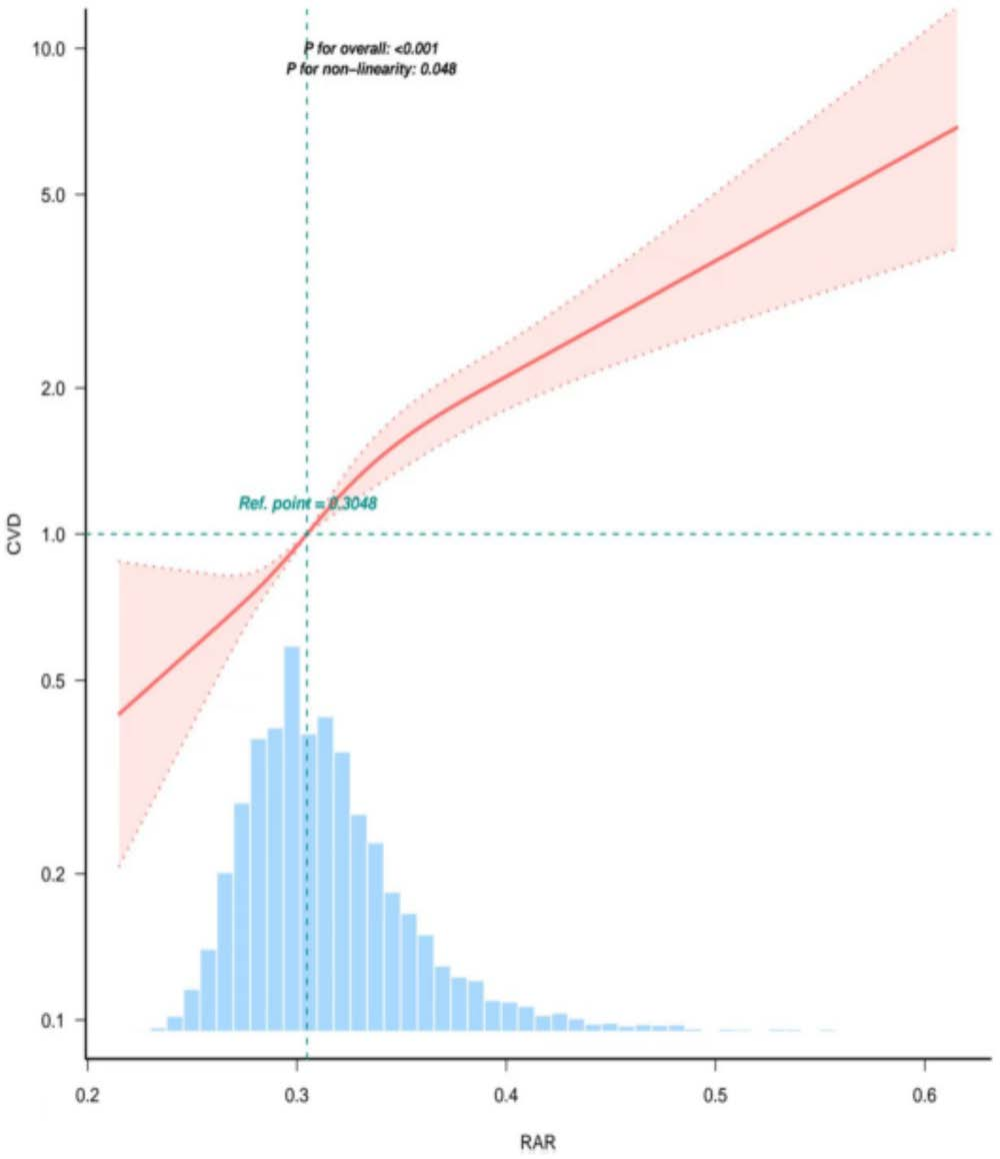
Curve fitting.

In the RCS, Figure 2 RAR and CVD risk have a nonlinear association. The reference point was set at RAR = 0.3048, above which the risk of CVD increases significantly. The *P*-value for the overall association is <.001, confirming the strong relationship. Notably, the *P*-value for nonlinearity is .048, indicating a significant nonlinear component to this relationship. Table 4 presents the analysis of the threshold effects of RAR in relation to CVD. The adjusted model showed that RAR < 0.416 was a critical threshold at which the ratio (OR) was extremely high at 9771.155 (95% CI: 1491.836–63,998.653, *P* < .001), indicating a strong association with CVD. Beyond this threshold, the OR decreased to 0 (95% CI: 0–0.453), indicating no significant association with CVD (*P* = .0357).

**Table 4:** The following study will examine the threshold effect of RAR in relation to CVD.

RAR	Adjusted model	
	OR (95% CI)	*P* value
<0.416	9771.155 (1491.836–63,998.653)	<.001
≥0.416	0 (0–0.453)	.0357
Log-likelihood ratio test	–	<.001

CI = confidence interval, CVD = cardiovascular disease, OR = odds ratio.

### 3.5. Stratified analyses based on additional variables

**Figure 3.**
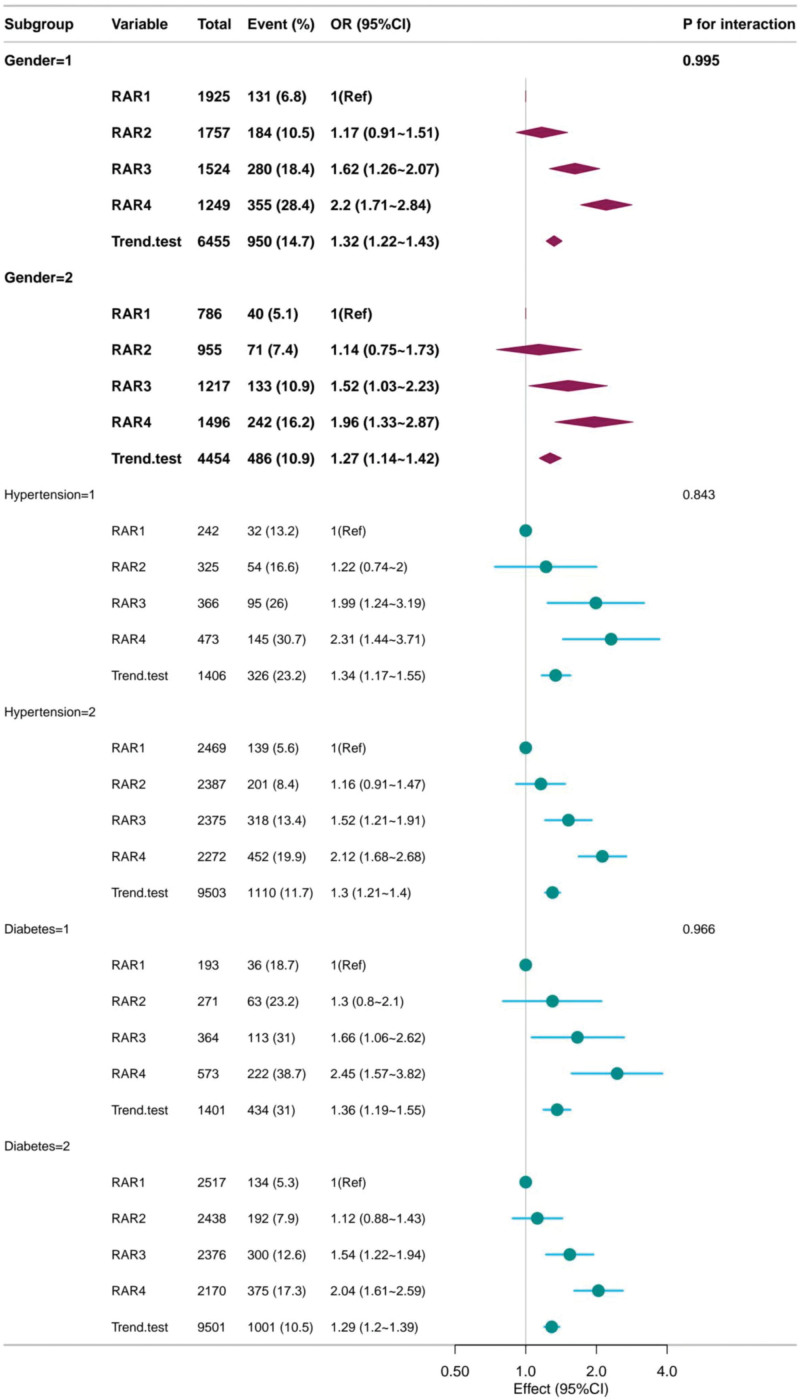
Forest map.

The forest plot in Figure 3 assesses the relationship between RAR and CVD risk in different subgroups, including sex, hypertensive status, and diabetic status. Interaction *P*-values showed no significant interaction between RAR category and subgroup variables (all *P* > .05), indicating that there was a constant connection between RAR and CVD risk across subgroups.

## 4. Discussion

This investigation represents the inaugural effort to examine the association between RAR and CVD. The study is based on the analysis of data from 10,909 individuals. Logistic regression analysis identified RAR as an independent risk factor for CVD, with all *P*-values being <.001. RCS analysis revealed a nonlinear relationship between RAR and CVD. Collectively, the results of the subgroup and interaction analyses indicate that the trends observed across all subgroups were consistent with those of the overall population.

The evidence presented in this study leads to the following conclusion: There is a nonlinear association between RAR and CVD among US adults. Adjusted modeling identified a critical threshold for RAR at 0.416, below which the odds ratio (OR) for CVD was exceedingly high at 9771.155 (95% CI: 1491.836–63,998.653, *P* < .001). This finding indicates a robust association between RAR and CVD. Clinicians may utilize this insight to predict and assess the likelihood of cardiovascular events through a stratified or dynamic management approach. Given the numerous advantages of RAR, its application in clinical settings appears to be a promising prospect.

CVD includes acute coronary syndrome, heart failure, and other CVDs.^[18]^ Acute coronary syndrome, the most predominant symptom is chest pain, and the most common concurrent symptoms with chest pain are dyspnea, sweating, unusual fatigue, nausea, and lightheadedness. The most predominant symptom of heart failure manifests as dyspnea (also known as shortness of breath, dyspnea, or breathlessness), and initially there may be stomach upset, nausea and vomiting, and loss of appetite.^[19]^ It may also manifest as fatigue^[20]^ and cognitive-kinetic impairment.

Abnormal leukocyte function and pro-inflammatory cytokines are central to CVD disease, leading to vascular dysfunction, impaired inflammatory abatement and promotion of chronic inflammation.^[21]^ It is evident that chronic inflammation plays a pivotal role in the development and progression of atherosclerotic thrombosis.^[22]^ This underscores the pivotal role of inflammation in the pathogenesis of CVD. To advance the diagnosis and management of CVD, there is a pressing need to develop and incorporate novel biomarkers that offer greater sensitivity and specificity into clinical practice. The immediate priority is to identify effective, simple, and stable novel inflammatory biomarkers that can enhance the early detection and risk stratification of CVD.

RAR is an emerging complex biomarker that demonstrates significant potential for predicting inflammation. Observations indicate that RAR increases in response to inflammatory stimuli. This elevation is believed to reflect the body’s inflammatory response, with higher RAR levels serving as a quantitative indicator of the magnitude and duration of inflammation. As inflammation progresses or intensifies, RAR levels correspondingly rise, thereby offering clinicians a dynamic measure of the body’s inflammatory burden.^[23]^ The association between elevated RDW and adverse outcomes in various inflammation-related diseases has garnered significant attention in the medical literature. Previous research has established that RDW is closely linked to the presence of numerous inflammatory conditions. An increase in RDW levels has been shown to correlate with a higher prevalence of these diseases, particularly among patients with cardiovascular disorders. Specifically, elevated RDW has been implicated in an increased incidence of mortality, heart failure, rheumatic diseases, and asthma in this patient population.^[24]^

A potential mechanism underlying the observed phenomena in CVD may involve the interplay of inflammation, hypoxia, and endothelial dysfunction. These pathophysiological processes are known to disrupt normal erythropoiesis and/or red blood cell survival, thereby contributing to elevated levels of RDW. Albumin (ALB) serves as a biomarker of inflammation, and in the context of inflammatory states, increased capillary permeability leads to the extravasation of ALB, thereby expanding its volume of distribution and the interstitial space. Additionally, the half-life of ALB is shortened, resulting in a reduction in its total circulating volume. Collectively, these alterations culminate in hypoalbuminemia. Notably, myocardial infarction, coronary artery disease, and ST-segment elevation myocardial infarction have been identified as conditions associated with alterations in albumin levels.^[25,26]^ The HALP score can represent a symptom marker, and ALB is one of its scores.^[27]^ Lower serum albumin levels promote chronic low-grade inflammation by impairing the body’s ability to clear inflammatory cytokines (TNF-α, IL-6) and free radicals and damaging the body’s binding and.^[28]^

Consequently, the manifestation of hypoalbuminemia may serve as an indicator of underlying inflammation. Given the established association between ALB and CVD, it is reasonable to hypothesize that RDW may also be correlated with CVD. As a leading cause of mortality among adults, CVD is significantly influenced by hyperlipidemia, with affected individuals exhibiting approximately a twofold increased risk of developing CVD compared to those with normal total cholesterol levels. Our study revealed a positive correlation between elevated RDW levels and an increased risk of CVD, thereby highlighting the potential importance of RDW reduction as a therapeutic target in the management of CVD.^[29]^ CVD remains the predominant cause of mortality among adults. Notably, individuals with hyperlipidemia exhibit an approximately twofold increased risk of developing CVD compared to those with normal total cholesterol levels. In our study, we observed a significant association between elevated RDW levels and an increased risk of CVD. These findings underscore the potential importance of RDW as a prognostic marker and highlight the need for further investigation into the therapeutic implications of targeting RDW reduction in the management of CVD.

The present study leverages a large and representative dataset, which is readily accessible and dynamically updated biennially, thereby ensuring the robustness and reliability of the findings. Moreover, we employed a comprehensive suite of statistical methods, including multivariate analyses, subgroup analyses, and interaction analyses, to rigorously control for potential confounding factors and enhance the validity of our results. In contrast to prior investigations, our study corroborates the established prognostic significance of RDW in various CVDs. However, our analysis extends beyond previous work by meticulously examining the nonlinear relationships between RDW and CVD risk. This detailed exploration provides novel insights into the underlying mechanisms through which RDW influences cardiovascular outcomes.

This study utilized a large and representative dataset, readily accessible and dynamically updated every 2 years, thereby ensuring the robustness and reliability of the findings. Furthermore, we employed a comprehensive set of statistical methods, including multivariate analysis, subgroup analysis, and interaction analysis, to rigorously control for potential confounding factors and enhance the validity of the results. Compared with prior studies, our research confirms the established prognostic significance of the red cell distribution width to albumin ratio (RAR) across various CVDs. However, our investigation further revealed a non-linear relationship between RAR and CVD risk. This detailed exploration provides novel insights into the potential mechanisms through which RAR influences cardiovascular outcomes.

Determining the critical threshold for the ratio of RDW to albumin (RAR) associated with CVD risk holds significant theoretical and practical implications. This finding not only provides a potential foundation for developing targeted therapeutic strategies aimed at modulating the RAR level to reduce CVD risk, but also underscores the importance of incorporating the RAR into routine clinical assessments for CVD. Whilst our findings are compelling, this study is not without limitations. The cross-sectional design precludes establishing causality, and the generalizability of results may be restricted to the specific cohort examined. Our study identifies the RAR as a significant predictor of CVD risk, particularly given its non-linear relationship with disease outcomes. The intrinsic interactions between the RAR and other cardiovascular risk factors warrant further investigation. Future research should prioritize elucidating the potential mechanisms through which the RAR influences CVD risk, and validate these findings in prospective studies. Furthermore, exploring the potential of the RAR as a therapeutic target represents a promising avenue for future investigation.

## 5. Reach a verdict

This study reveals a noteworthy clinically relevant finding: a non-linear relationship exists between the ratio of RAR and CVD risk. Regardless of whether RAR is analyzed as a categorical or continuous variable, higher RAR values correlate with an increased risk of CVD. For instance, given its potential impact on overall cardiovascular health, appropriate serum albumin supplementation must be provided when low serum albumin levels are detected. In cases where an elevated RAR is observed, it is imperative to conduct a thorough investigation to determine the underlying etiology and initiate targeted interventions aimed at normalizing RAR levels, whilst simultaneously minimizing the associated risks currently attributable to the RAR.

## Acknowledgments

We would like to thank all participants in this study.

## Author contributions

**Conceptualization**: Siqi Ma, Qu Jin, Jingyi Yang.

**Data curation**: Siqi Ma, Qu Jin, Jingyi Yang.

**Formal analysis**: Siqi Ma.

**Funding acquisition**: Siqi Ma.

**Investigation**: Liping Chang, Zhuo Yan.

**Methodology**: Liping Chang, Zhuo Yan.

**Project administration**: Liping Chang.

**Resources**: Liping Chang.

**Writing – original draft**: Siqi Ma, Qu Jin, Zhuo Yan.

**Writing – review & editing**: Siqi Ma.

## References

[1] Katta N Loethen T Lavie CJ Alpert MA. Obesity and coronary heart disease: epidemiology, pathology, and coronary artery imaging. Curr Probl Cardiol. 2021;46:100655. 32843206 10.1016/j.cpcardiol.2020.100655

[2] Roth GA Mensah GA Johnson CO; GBD-NHLBI-JACC Global Burden of Cardiovascular Diseases Writing Group. Global burden of cardiovascular diseases and risk factors, 1990–2019: update from the GBD 2019 study. J Am Coll Cardiol. 2020;76:2982–3021. 33309175 10.1016/j.jacc.2020.11.010 PMC7755038

[3] Ruan L Xu S Qin Y. Red blood cell distribution width to albumin ratio for predicting type I cardiorenal syndrome in patients with acute myocardial infarction: a retrospective cohort study. J Inflamm Res. 2024;17:3771–84. 38882186 10.2147/JIR.S454904 PMC11180445

[4] Wang J Zhang Y Wan Y Fan Z Xu R. The relationship between red blood cell distribution width and incident diabetes in Chinese adults: a cohort study. J Diabetes Res. 2020;2020:1623247. 32185232 10.1155/2020/1623247 PMC7063217

[5] Lan W Liu E Sun D. Red cell distribution in critically ill patients with chronic obstructive pulmonary disease. Pulmonology. 2024;30:34–42. 35501276 10.1016/j.pulmoe.2022.04.001

[6] Saad E Maamoun B Nimer A. Increased red blood cell distribution predicts severity of chronic obstructive pulmonary disease exacerbation. J Pers Med. 2023;13:843. 37241013 10.3390/jpm13050843 PMC10222320

[7] Xie KH Liu L-L Liang Y-R. Red cell distribution width: a novel predictive biomarker for stroke risk after transient ischaemic attack. Ann Med. 2022;54:1167–77. 35471128 10.1080/07853890.2022.2059558 PMC9045760

[8] Song B Shi P Xiao J. Utility of red cell distribution width as a diagnostic and prognostic marker in non-small cell lung cancer. Sci Rep. 2020;10:15717. 32973271 10.1038/s41598-020-72585-4 PMC7515922

[9] Manolis AA Manolis TA Melita H Mikhailidis DP Manolis AS. Low serum albumin: a neglected predictor in patients with cardiovascular disease. Eur J Intern Med. 2022;102:24–39. 35537999 10.1016/j.ejim.2022.05.004

[10] Liu Y Sun S Liu L. Association between the red blood cell distribution width-albumin ratio and cardiovascular diseases. Front Cardiovasc Med. 2025;12:1529533. 40271132 10.3389/fcvm.2025.1529533 PMC12014755

[11] Li D Ruan Z Wu B. Association of red blood cell distribution width-albumin ratio for acute myocardial infarction patients with mortality: a retrospective cohort study. Clin Appl Thromb Hemost. 2022;28:10760296221121286. 36045634 10.1177/10760296221121286 PMC9445528

[12] Zhou P Hao Z Chen Y Zhang Z Xu W Yu J. Association between gut microbiota and diabetic microvascular complications: a two-sample Mendelian randomization study. Front Endocrinol (Lausanne). 2024;15:1364280. 39157683 10.3389/fendo.2024.1364280 PMC11327146

[13] Liu J Wang X Gao TY. Red blood cell distribution width to albumin ratio associates with prevalence and long-term diabetes mellitus prognosis: an overview of NHANES 1999–2020 data. Front Endocrinol (Lausanne). 2024;15:1362077. 39114290 10.3389/fendo.2024.1362077 PMC11303207

[14] Zhao F Liu M Kong L. Association between red blood cell distribution width-to-albumin ratio and diabetic retinopathy. J Clin Lab Anal. 2022;36:e24351. 35285094 10.1002/jcla.24351 PMC8993659

[15] Eyiol A Ertekin B. Association of red blood cell distribution width to albumin ratio with prognosis in stroke patients. Biomark Med. 2024;18:311–20. 38648096 10.2217/bmm-2023-0460 PMC11218802

[16] Yin L Min J Zhong L Shen Q. The correlation between red cell distribution width to albumin ratio and all-cause mortality in critically ill patients with rheumatic diseases: a population-based retrospective study. Front Med (Lausanne). 2023;10:1199861. 37908850 10.3389/fmed.2023.1199861 PMC10614050

[17] Dodge LE Phillips SJ Neo DT Nippita S Paul ME Hacker MR. Quality of information available online for abortion self-referral. Obstet Gynecol. 2018;132:1443–52. 30399097 10.1097/AOG.0000000000002950 PMC6249052

[18] Yang Q Zheng J Chen W. Association between preadmission metformin use and outcomes in intensive care unit patients with sepsis and type 2 diabetes: a cohort study. Front Med (Lausanne). 2021;8:640785. 33855034 10.3389/fmed.2021.640785 PMC8039324

[19] Jurgens CY Lee CS Aycock DM; American Heart Association Council on Cardiovascular and Stroke Nursing; Council on Hypertension; and Stroke Council. State of the science: the relevance of symptoms in cardiovascular disease and research: a scientific statement from the American Heart Association. Circulation. 2022;146:e173–84. 35979825 10.1161/CIR.0000000000001089

[20] Valentova M von Haehling S Bauditz J. Intestinal congestion and right ventricular dysfunction: a link with appetite loss, inflammation, and cachexia in chronic heart failure. Eur Heart J. 2016;37:1684–91. 26865478 10.1093/eurheartj/ehw008

[21] AbouEzzeddine OF Wong YW Mentz RJ; NHLBI Heart Failure Clinical Research Network. Evaluation of novel metrics of symptom relief in acute heart failure: the worst symptom score. J Card Fail. 2016;22:853–8. 26718344 10.1016/j.cardfail.2015.12.015 PMC4916018

[22] Estruch R Sacanella E Lamuela-Raventos RM. Ideal dietary patterns and foods to prevent cardiovascular disease: beware of their anti-inflammatory potential. J Am Coll Cardiol. 2020;76:2194–6. 33153577 10.1016/j.jacc.2020.09.575

[23] Salvagno GL Sanchis-Gomar F Picanza A Lippi G. Red blood cell distribution width: a simple parameter with multiple clinical applications. Crit Rev Clin Lab Sci. 2015;52:86–105. 25535770 10.3109/10408363.2014.992064

[24] Ding J Zhang Y Chen X. Red cell distribution width to albumin ratio is associated with asthma risk: a population-based study. Front Med (Lausanne). 2024;11:1493463. 39722824 10.3389/fmed.2024.1493463 PMC11668568

[25] Chen Y Guan M Wang R Wang X. Relationship between advanced lung cancer inflammation index and long-term all-cause, cardiovascular, and cancer mortality among type 2 diabetes mellitus patients: NHANES, 1999–2018. Front Endocrinol (Lausanne). 2023;14:1298345. 38111710 10.3389/fendo.2023.1298345 PMC10726345

[26] Karakayali M Omar T Artac I. The prognostic value of HALP score in predicting in-hospital mortality in patients with ST-elevation myocardial infarction undergoing primary percutaneous coronary intervention. Coron Artery Dis. 2023;34:483–8. 37799045 10.1097/MCA.0000000000001271

[27] Zhang T Liu P. Association of hemoglobin, albumin, lymphocyte, and platelet score with all-cause and cardiovascular mortality in adults with diabetes or prediabetes: results from NHANES 2005–2018. Prev Med Rep. 2025;54:103101. 40469253 10.1016/j.pmedr.2025.103101 PMC12136913

[28] Cabrerizo S Cuadras D Gomez-Busto F Artaza-Artabe I Marín-Ciancas F Malafarina V. Serum albumin and health in older people: review and meta analysis. Maturitas. 2015;81:17–27. 25782627 10.1016/j.maturitas.2015.02.009

[29] Schalk BW Visser M Bremmer MA Penninx BWJH Bouter LM Deeg DJH. Change of serum albumin and risk of cardiovascular disease and all-cause mortality: longitudinal aging study Amsterdam. Am J Epidemiol. 2006;164:969–77. 16980573 10.1093/aje/kwj312

